# Association of hyperchloremia with all-cause mortality in patients admitted to the surgical intensive care unit: a retrospective cohort study

**DOI:** 10.1186/s12871-021-01558-5

**Published:** 2022-01-07

**Authors:** Keke Song, Tingting Yang, Wei Gao

**Affiliations:** Department of Anesthesiology, The First Affiliated Hospital of Xi’an Jiaotong University, No. 277, Yanta West Road, Yanta District, Xi’an, China

**Keywords:** Hyperchloremia, All-cause mortality, Surgical intensive care unit

## Abstract

**Background:**

Serum chloride (Cl^−^) is one of the most essential extracellular anions. Based on emerging evidence obtained from patients with kidney or heart disease, hypochloremia has been recognized as an independent predictor of mortality. Nevertheless, excessive Cl^−^ can also cause death in severely ill patients. This study aimed to investigate the relationship between hyperchloremia and high mortality rate in patients admitted to the surgical intensive care unit (SICU).

**Methods:**

We enrolled 2131 patients from the Multiparameter Intelligent Monitoring in Intensive Care III database version 1.4 (MIMIC-III v1.4) from 2001 to 2012. Selected SICU patients were more than 18 years old and survived more than 72 h. A serum Cl^−^ level ≥ 108 mEq/L was defined as hyperchloremia. Clinical and laboratory variables were compared between hyperchloremia (*n* = 664) at 72 h post-ICU admission and no hyperchloremia (*n* = 1467). The Locally Weighted Scatterplot Smoothing (Lowess) approach was utilized to investigate the correlation between serum Cl- and the thirty-day mortality rate. The Cox proportional-hazards model was employed to investigate whether serum chlorine at 72 h post-ICU admission was independently related to in-hospital, thirty-day and ninety-day mortality from all causes. Kaplan-Meier curve of thirty-day and ninety-day mortality and serum Cl^−^ at 72 h post-ICU admission was further constructed. Furthermore, we performed subgroup analyses to investigate the relationship between serum Cl^−^ at 72 h post-ICU admission and the thirty-day mortality from all causes.

**Results:**

A J-shaped correlation was observed, indicating that hyperchloremia was linked to an elevated risk of thirty-day mortality from all causes. In the multivariate analyses, it was established that hyperchloremia remained a valuable predictor of in-hospital, thirty-day and ninety-day mortality from all causes; with adjusted hazard ratios (95% CIs) for hyperchloremia of 1.35 (1.02 ~ 1.77), 1.67 (1.28 ~ 2.19), and 1.39 (1.12 ~ 1.73), respectively. In subgroup analysis, we observed hyperchloremia had a significant interaction with AKI (P for interaction: 0.017), but there were no interactions with coronary heart disease, hypertension, and diabetes mellitus (P for interaction: 0.418, 0.157, 0.103, respectively).

**Conclusion:**

Hyperchloremia at 72 h post-ICU admission and increasing serum Cl^−^ were associated with elevated mortality risk from all causes in severely ill SICU patients.

Serum chloride (Cl^−^) is one of the essential extracellular anions responsible for about a third of plasma tonicity, about 97-98% of all the strong anionic charges, and two-thirds of negative charges in the plasma [1, 2]. Cl^−^ plays pivotal roles in numerous body functions, e.g., maintenance of acid-base balance, maintenance of osmotic pressure, maintenance of muscular activity, and the movement of water between fluid compartments [1]. Despite its physiologic importance, it might still be easier to focus on potassium or sodium and water balance rather than Cl^−^ in clinical settings. Much less research attention has been accorded to Cl^−^ relative to other electrolytes. However, recently, more and more studies have reported the significance of serum Cl^−^. Emerging research evidence on individuals with heart or kidney disease has shown that hypochloremia served as an independent indicator of mortality [3–7]. However, hyperchloremia is also related to poor clinical outcomes in severely ill patients [8–11].

Nevertheless, to our knowledge, no study has studied the relationship linking hyperchloremia to mortality in the surgical intensive care unit (SICU). To that end, we aimed to establish whether hyperchloremia is linked to a high mortality rate in patients admitted to the SICU.

## Materials and methods

This involved a retrospective cohort study that employed Multiparameter Intelligent Monitoring in Intensive Care III database version 1.4 (MIMIC-III v1.4). From 2001 to 2012, more than 38,000 patients in the Beth Israel Deaconess Medical Center’s ICU in Boston, Massachusetts, United States (US) have been included in the MIMIC-III v1.4 [2, 12]. The database is publicly available to researchers who have completed a ‘protecting human subjects’ training. The Review Board of the Massachusetts Institute of Technology and Beth Israel Deaconess Medical Center approved the establishment of the database. Thus, consent was obtained for the original data collection, but not specifically for this study. Data presented in this study were extracted by the author, Song, who completed the online training course of the National Institutes of Health (certification number: 37814178). The data was extracted using the PostgreSQL tool V.9.6 to obtain the clinical data containing patient demographics, laboratory findings, mortality, and other clinical variables. To maintain the confidentiality of the included study subjects, all sensitive information was hidden.

### Population selection criteria

The MIMIC-III v1.4 comprised of 46,476 patients. Patients were selected according to the following inclusion criteria: 1) first hospitalization; 2) admitted to the SICU; and 3) older than 18. Participants were excluded according to the following criteria: 1) no serum Cl^−^ at 72 h after ICU admission; 2) missing > 5% individual data.

### Data abstraction

The demographic factors, laboratory parameters, the scoring system, and the clinical factors were extracted. Only the data of the first SICU admission of each study subject were utilized, and baseline data were abstracted within 24 h after SICU admission. The comorbidities consisted of coronary heart disease (diagnosed as “coronary atherosclerosis” in the database), chronic obstructive pulmonary disease (COPD, diagnosed as “obstructive chronic bronchitis without exacerbation” or “obstructive chronic bronchitis with (acute) exacerbation” or “obstructive chronic bronchitis with acute bronchitis” in the database), hypertension (diagnosed as “benign essential hypertension” or “unspecified essential hypertension” in the database), diabetes mellitus (diagnosed as “diabetes” in the database), and acute kidney injury (AKI, an increase in serum creatinine level of more than 1.5 times above baseline). Laboratory findings at 72 h after ICU admission included serum chloride (Cl_72h_), serum sodium (Na_72h_), serum potassium (K_72h_), blood urea nitrogen (BUN_72h_), serum creatinine (Scr_72h_), white blood cells (WBC_72h_), platelet (PLT_72h_), and hemoglobin and serum chloride at SICU admission (Cl_0_). Systemic Inflammatory Response Syndrome (SIRS) score, Elixhauser comorbidity index, Sequential Organ Failure Assessment (SOFA) score, as well as Simplified Acute Physiology Score II (SAPS II), were acquired at 72 h after ICU admission. Moreover, survival data on the vital status was retrieved from the US Social Security death index records. In-hospital, thirty-day and ninety-day mortality constituted the endpoints for this analysis.

### Statistical analysis

The sample was classified into two subgroups based on serum Cl^−^ levels at 72 h after ICU admission: hyperchloremia (Cl _72h_ ≥ 108 mmol/L) and no hyperchloremia (Cl _72h_ < 108 mmol/L). The baseline features of all the study subjects were stratified based on these two groups; with continuous variables indicated as mean ± standard deviation (SD) or interquartile range (IQR) and median. Summaries of the categorical data are indicated as the percentage or number. The chi-squared test was employed to compare the categorical data. The Lowess smoothing method was employed to explore the association of serum Cl^−^ with thirty-day mortality. To facilitate the clinical interpretation of our findings, we employed the Cox proportional hazards models to establish if serum Cl_72h_ was independently linked to in-hospital, thirty-day and ninety-day mortality from all causes, with findings expressed as hazard ratios (HRs) with 95% confidence intervals (CIs). We applied 2 multivariate models for all the endpoints. The no hyperchloremia group served as the control group. In model I, we adjusted covariates for age, ethnicity and male. In model II, we adjusted the covariates for age, ethnicity, male, SAPS II, renal replacement therapy (RRT), Cl_0_, Scr_72h_, bicarbonate_72h_, PLT_72h_. These confounders were selected based on a change in effect estimate of more than 10%.

Subgroup analyses were carried out to probe the interaction linking serum Cl _72h_ with thirty-day all-cause mortality, consisting of coronary heart disease, hypertension, diabetes mellitus and acute kidney injury, Adjusted by age, ethnicity, male, SAPS II, RRT, Cl_0_, Scr_72h_, bicarbonate_72h_, PLT_72h_. The R software V.3.42 (The R Project for Statistical Computing, Vienna, Austria) was utilized for data analysis. *P*-value < 0.05 represents statistical significance, with all reported *P*-values being two-sided.

## Results

### Participant characteristics

**Fig. 1:**
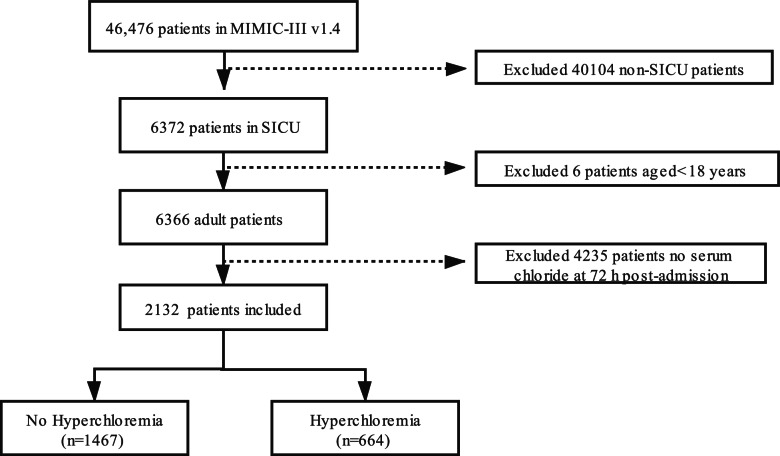
Flow chart illustrating patient selection. MIMIC-III v1.4 Multiparameter Intelligent Monitoring in Intensive Care III database version 1.4, SICU surgical intensive care unit

Overall, 2131 subjects were enrolled based on the inclusion criteria and were clustered into two groups. The study sample was divided into two subgroups according to the serum chloride levels at 72 h after ICU admission: *n* = 664 subjects in the hypochloremia group and *n* = 1467 subjects in the no hyperchloremia group (Fig. 1). The characteristics of the subjects based on serum Cl^−^ contents at 72 h after ICU admission are shown in Table 1. We observed that white male subjects exhibited a higher SICU hospitalization rate. Moreover, participants with hyperchloremia were more likely to be older, had higher SIRS, SOFA and SAPS II scores. Furthermore they also had higher admission serum chloride, sodium and sodium at 72 h after admission to ICU, longer length of ICU stay and hospital stay.

**Table 1:** Clinical Features Stratified by Serum Chloride at 72 h post-ICU admission

Variable	Total(*n* = 2131)	No Hyperchloremia (Cl_72h_ < 108, *n* = 1467)	Hyperchloremia (Cl_72h_ ≥ 108, *n* = 664)	*P*-value
Age, yr, median (IQR)	64.0 (52.0-77.0)	63.0 (52.0-76.0)	67.0 (53.0-78.2)	< 0.001
Male, n(%)	1110 (52.1%)	787 (53.6%)	323 (48.6%)	0.032
Ethnicity, n(%)				< 0.001
White	1565 (73.4%)	1114 (75.9%)	451 (67.9%)	
Black	182 (8.5%)	120 (8.2%)	62 (9.3%)	
Asian	73 (3.4%)	46 (3.1%)	27 (4.1%)	
Other	311 (14.6%)	187 (12.7%)	124 (18.7%)	
Comorbidities, n (%)				
Coronary heart disease	246 (11.5%)	170 (11.6%)	76 (11.4%)	0.924
COPD	29 (1.4%)	22 (1.5%)	7 (1.1%)	0.411
Hypertension	982 (46.1%)	669 (45.6%)	313 (47.1%)	0.51
Diabetes mellitus	544 (25.5%)	383 (26.1%)	161 (24.2%)	0.362
Acute kidney injury	508 (23.8%)	343 (23.4%)	165 (24.8%)	0.461
Scoring systems, median (IQR)				
SIRS	3.0 (2.0-4.0)	3.0 (2.0-4.0)	3.0 (2.0-4.0)	0.005
SOFA	4.0 (2.0-6.0)	3.0 (2.0-6.0)	4.0 (2.0-6.0)	< 0.001
SAPSII	35.0 (26.0-44.0)	33.0 (25.0-43.0)	37.0 (29.0-47.0)	< 0.001
Elixhauser comorbidity	11.0 (1.0-20.0)	11.0 (0.0-20.0)	11.0 (3.0-20.0)	0.06
Therapeutic exposure in ICU,n(%)				
Renal replacement therapy	90 (4.2%)	77 (5.2%)	13 (2.0%)	< 0.001
Diuretic	1052 (49.4%)	708 (48.3%)	344 (51.8%)	0.129
Statin	684 (32.1%)	459 (31.3%)	225 (33.9%)	0.234
Pressin	161 (7.6%)	99 (6.7%)	62 (9.3%)	0.036
Plasmalyte	6 (0.3%)	4 (0.3%)	2 (0.3%)	0.908
Fluid balance within 72 h after ICU admission(mL), median (IQR)	− 2176.0 (− 6688.0- 3296.0)	− 1977.0 (− 6284.0, 2945.0)	− 2980.0 (− 7464.0, 4446.0)	0.203
Admission serum bicarbonate(mmol/L), median (IQR)	24.0 (21.0-26.0)	24.0 (22.0-27.0)	23.0 (20.0-25.0)	< 0.001
Serum bicarbonate at 72 h after ICU admission(mmol/L), median (IQR)	25.0 (23.0-28.0)	26.0 (24.0-28.0)	23.0 (20.0-25.0)	< 0.001
Admission serum Cl^−^(mmol/L), median (IQR)	105.0 (101.0-108.0)	104.0 (101.0-107.0)	107.5 (104.0-111.0)	< 0.001
Admission serum sodium (mmol/L), median (IQR)	139.0 (136.0-141.0)	138.0 (135.0-140.0)	140.0 (138.0-142.0)	< 0.001
Serum sodium at 72 h after ICU admission(mmol/L), median (IQR)	139.0 (137.0-142.0)	138.0 (136.0-140.0)	143.0 (141.0-146.0)	< 0.001
Length of ICU stay, d, median (IQR)	5.0 (2.0-9.0)	4.0 (2.0-8.0)	7.0 (4.0-13.0)	< 0.001
Length of hospital stay, d, median (IQR)	12.0 (8.0-19.0)	11.0 (7.0-18.0)	14.0 (8.0-22.0)	< 0.001

*ICU* intensive care unit, *IQR* interquartile range, *COPD* chronic obstructive pulmonary disease, *SIRS* Systemic Inflammatory Response Syndrome, *SOFA* Sequential Organ Failure Assessment, *SAPS II* Simplified Acute Physiology Score II

### Chloride levels and clinical endpoints

**Fig. 2:**
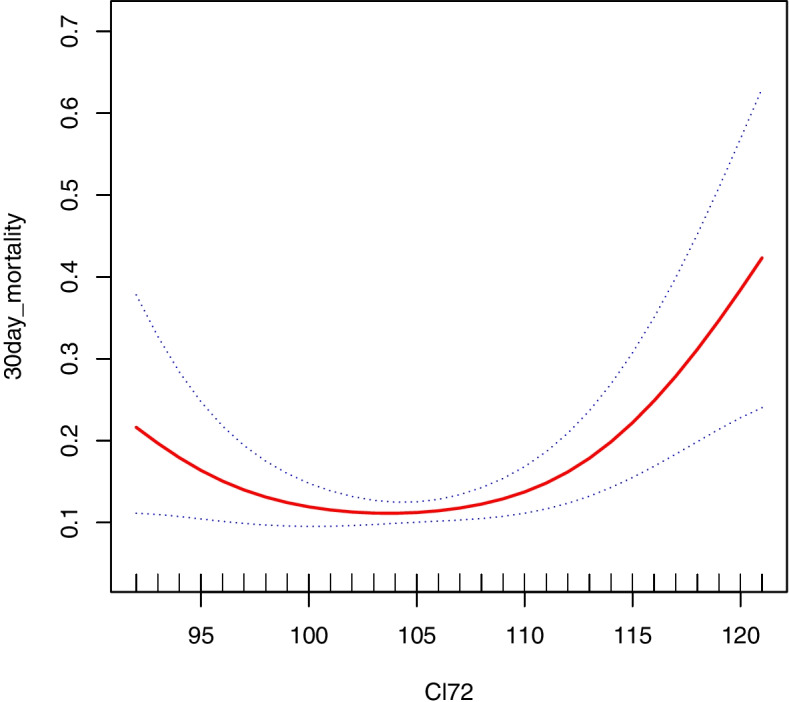
Relationship between serum chloride at 72 h after ICU admission and 30-day all-cause mortality. *Cl*_*72*_ serum chloride at 72 h after ICU admission

Figure 2 indicates that Cl_72h_ was non-linearly linked to the thirty-day mortality from all causes. A J-shaped association was reported, demonstrating that hyperchloremia was associated with an elevated risk of thirty-day mortality from all causes. Multivariate analysis adjusted for age, male, and ethnicity demonstrated that hyperchloremia were good predictors of risk for in-hospital, thirty-day and ninety-day mortality from all causes, with adjusted HRs (95% CIs) for hyperchloremia being 1.32 (1.04 ~ 1.69), 1.68 (1.32 ~ 2.12), and 1.51 (1.24 ~ 1.83), respectively. After adjusting for age, ethnicity, male, SAPS II, RRT, Cl_0_, Scr_72h_, bicarbonate_72h_, PLT_72h_, we established that hyperchloremia was still a reliable risk predictor for in-hospital, thirty-day and ninety-day mortality from all causes, and adjusted HRs (95% CIs) for hyperchloremia were 1.35 (1.02 ~ 1.77), 1.67 (1.28 ~ 2.19), and 1.39 (1.12 ~ 1.73), respectively (Table 2).

**Table 2:** Hazard ratios and 95% confidence intervals for mortality across groups of serum chloride at 72 h after ICU admission

Chloride	Non-adjusted		Model I		Model II	
	HR (95% CI)	*P*-value	HR (95% CI)	*P*-value	HR (95% CI)	*P*-value
Hospital all-cause mortality						
fitted groups, mmol/L						
< 108	1.0 (ref)		1.0 (ref)		1.0 (ref)	
≥ 108	1.47 (1.15, 1.87)	0.002	1.32 (1.04 ~ 1.69)	0.025	1.35 (1.02 ~ 1.77)	0.035
30-day all-cause mortality						
fitted groups, mmol/L						
< 108	1.0 (ref)		1.0 (ref)		1.0 (ref)	
≥ 108	1.86 (1.47, 2.35)	< 0.001	1.68 (1.32 ~ 2.12)	< 0.001	1.67 (1.28 ~ 2.19)	< 0.001
90-day all-cause mortality						
fitted groups, mmol/L						
< 108	1.0 (ref)		1.0 (ref)		1.0 (ref)	
≥ 108	1.64 (1.35, 1.98)	< 0.001	1.51 (1.24 ~ 1.83)	< 0.001	1.39 (1.12 ~ 1.73)	0.003

Models were derived using Cox proportional hazards regression models

Non-adjusted model adjusted for: none

Adjust I model adjust for: age, ethnicity, male

Adjust II model adjust for: age, ethnicity, male, SAPS II, RRT, Cl_0_, Scr_72h_, bicarbonate_72h_, PLT_72h_

*HR* hazard ratio, *CI* confidence interval, *SAPS II* Simplified Acute Physiology Score II, *RRT* renal replacement therapy, *Cl*_*0*_ admission serum chloride, *Scr*_*72h*_ creatinine_72h_, *PLT*_*72h*_ platelet_72h_

**Fig. 3:**
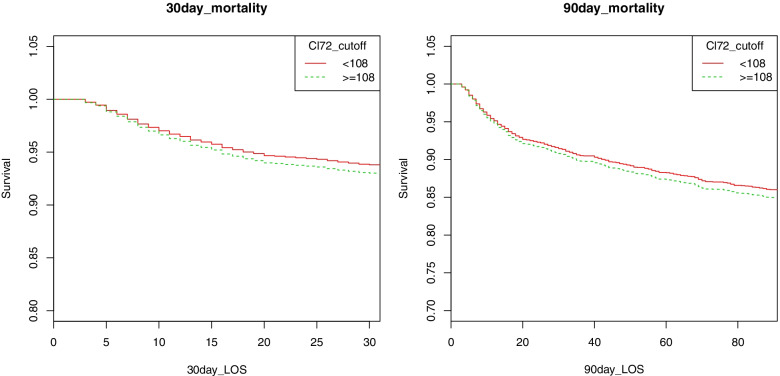
Kaplan-Meier curve of 30-day and 90-day mortality serum chloride at 72 h after ICU admission. *ICU* intensive care unit, *Cl*_*72*_ serum chloride at 72 h after ICU admission, *LOS* long of survival

The Kaplan-Meier curve of thirty-day mortality based on serum Cl^−^ at 72 h post-ICU admission is shown in Fig. 3. Cumulative thirty-day survival rates at 72 h post-ICU admission were 84.97 and 86.01% in the hyperchloremia and no hyperchloremia groups, respectively (*P* = 0.0011). Meanwhile, cumulative ninety-day survival rates at 72 h post-ICU admission were 93.01 and 93.80% in the hyperchloremia and no hyperchloremia groups, respectively (*P* = 0.0098) (Fig. 3).

### Subgroup analyses

Subgroup analyses were carried out to investigate the relationship of serum Cl^−^ at 72 h post-ICU admission with the thirty-day mortality from all causes. There were no notable interactions with coronary heart disease, hypertension, and diabetes mellitus (P for interaction: 0.418, 0.157, 0.103, respectively). But the hyperchloremia had an interactions with AKI, the no AKI patient who had hyperchloremia would have a higher thirty-day mortality(P for interaction: 0.017) (Table 3).

**Table 3:** Subgroup Analysis of the Comorbidities between serum Chloride at 72 h after ICU Admission and 30 day All-cause Mortality

	N	No Hyperchloremia (Cl_72h_ < 108) HR (95% CI)	Hyperchloremia (Cl_72h_ ≥ 108) HR (95% CI)	*P* value	P for interaction
Coronary heart disease					0.418
No	1885	1.0 (ref)	1.69 (1.26 ~ 2.25)	< 0.001	
Yes	246	1.0 (ref)	2.17 (1.05 ~ 4.51)	0.037	
Hypertension					0.157
No	1149	1.0 (ref)	1.3 (0.89 ~ 1.9)	0.169	
Yes	982	1.0 (ref)	2.36 (1.61 ~ 3.48)	< 0.001	
Diabetes mellitus					0.103
No	1587	1.0 (ref)	1.93 (1.43 ~ 2.62)	< 0.001	
Yes	544	1.0 (ref)	1.25 (0.69 ~ 2.27)	0.465	
Acute kidney injury					0.017
No	1623	1.0 (ref)	2.22 (1.57 ~ 3.14)	< 0.001	
Yes	508	1.0 (ref)	1.06 (0.68 ~ 1.66)	0.794	

*ICU* intensive care unit, *Cl*_*72*_ serum chloride at 72 h after ICU admission

## Discussion

Herein, we reported a J-shaped association linking serum chloride levels at 72 h post-ICU admission to thirty-day mortality from all causes. After multivariate logistic regression assessment was adjusted for other significant variables, we found that hyperchloremia remained independently associated with in-hospital, thirty-day and ninety-day mortality from all causes in patients admitted to the SICU.

Our study contributes to the growing research evidence indicating that hyperchloremia might be harmful in specific inpatient populations. Similarly, a previous investigation reported hyperchloraemia at 48 h was markedly linked to AKI and mortality in a multidisciplinary intensive care unit [5]. Moreover, hyperchloremia 48 h post-admission along with Δ Cl^−^ (the delta of chloride between the ICU admission and 48 h) were related to thirty-day mortality in major trauma patients [8]. More and more studies have shown that hyperchloremia is linked to mortality after surgery [9, 10, 13, 14], with the possible cause being fluid replacement during surgery which often consists of serum with high chlorine content.

Moreover, after admission to the ICU, the possible cause of hyperchloraemia is intravenous infusion [15]. The most commonly used liquid for hospitalization is normal saline, and the average Cl^−^ concentration is 154 Eq/L, which is higher than the average normal plasma Cl^−^ concentration [16]. At the same time, normal saline is often used as a diluent for drugs and might be an unaccounted source of chloride in the ICU. A large sample-sized retrospective investigation from a single-center ICU showed that maintenance and replacement fluids markedly contributed to daily Cl^−^ intake relative to resuscitation fluids [17]. Hyperchloremia can be toxic to cells, causing unwarranted requirements on cellular energy metabolism [18], and contributes to additional morbidity and mortality [19]. Hence, caution should be taken to minimize serum chlorine overload [20].

Taken together, our study found that serum Cl^−^ content at 72 h post-admission was associated with mortality from all causes in severely ill patients admitted to the surgical intensive care unit. To our knowledge, this is the first study in a broad SICU population. The strengths of this study include its large sample size and the multivariate adjustment for clinical confounders directly related to hyperchloremia and hospital mortality, including SOFA, SAPS II score, SIRS, and AKI. No previous study has documented the relationship of serum Cl^−^ level with hospital mortality while also accounting for confounding factors. The multivariate design, along with the patient population, The multivariate design, along with the patient population contributed to the robustness of this study.

## Conclusion

We found that hyperchloremia at 72 h post-ICU admission and increasing serum chloride were associated with an elevated risk of mortality from all causes in critically ill SICU patients. However, further in-depth studies in larger prospective multi-centers are warranted to verify and substantiate our findings.

### Limitation

Nevertheless, our present study has several limitations. Firstly, this study is a single-center retrospective study that possesses inherent biases. Secondly, we collected data for serum Cl- in patients at 72 h post-ICU admission only and did not assess other time points during the SICU stay. Finally, although we applied a multivariate model to control bias, many other known and unknown factors remain.

## Data Availability

The datasets used and/or analyzed during the current study will be available from the corresponding author on reasonable request. However, reanalysis of the full data needs to be approved by the MIMIC III Institute.

## Abbreviations

SICU: Surgical intensive care unit
MIMIC-III v1.4: Multiparameter Intelligent Monitoring in Intensive Care III database version 1.4
ICU: Intensive care unit
Cl^−^: Chloride
Na: Sodium
K: Potassium
BUN: Blood urea nitrogen
Scr: Serum creatinine
WBC: White blood cells
SIRS: Systemic Inflammatory Response Syndrome
SOFA: Sequential Organ Failure Assessment
SAPS II: Simplified Acute Physiology Score II
AKI: Acute kidney injury
